# In the Footsteps of Pasteur: Identifying Conglomerate Systems Using State-of-the-Art Electron Diffraction

**DOI:** 10.1063/4.0000931

**Published:** 2025-10-27

**Authors:** Gustavo Santiso-Quinones, Christian Jandl, Ivo B. Rietveld, Felix Painsecq, Gerard Coquerel, Laura Samperisi, Johannes Merkelbach, Gunther Steinfeld, Danny Stam

**Affiliations:** 1 ELDICO Scientific AG, Allschwil, Basel Area, Switzerland; 2 Normandie University, Rouen, Normandy, France; 3 Université Paris Cité Cité, Paris, Paris, France

## Abstract

Chirality is a key concept in structural chemistry and of crucial importance whenever biological systems are concerned. Ever since Pasteur’s discovery of the phenomenon in 1848, the research of chiral compounds is closely related to crystallography and single crystal X-ray diffraction is currently the standard method for absolute structure determination of chiral molecules or chiral molecular assemblies. In Pasteur’s example of sodium ammonium tartare tetrahydrate, the racemic compound crystallizes as separate enantiopure crystals, a so-called conglomerate – which allowed him to inspect individual crystals under the microscope and manually separate them by recognising their hemihedral faces. Most racemates, however, simply form racemic crystals, so the identification of conglomerate systems is important as only this allows for chiral discrimination in the solid state, e.g. for separation and purification of chiral molecules and chiral supramolecular assemblies.

The advancement of electron diffraction (microED, 3D ED) makes it possible to identify conglomerates with an up-to-date version of Pasteur’s method. Diffraction data can be collected from individual crystals of a racemic powder sample and dynamical refinement allows the determination of their absolute structure. In this case study the method was applied to chiral benzyl (*p*-tolyl) sulfoxide and achiral benzyl (*p*-tolyl) sulfone, which crystallizes in chiral assemblies. In both cases the results unambiguously prove the presence of both enantiomorphs and therefore identify them as conglomerate systems. For the achiral sulfone, where chirality only exists in the solid state, this allowed the preparation of an enantiopure sample by Viedma ripening. With fast measurement times and the ability to work with powder samples, very small sample amounts and without any prior knowledge, electron diffraction has the potential to establish itself as a new standard method in screening for conglomerates.

